# Assessing Individuals Attending the Family Medicine Clinic for Periodic Health Examinations in Screening of Diabetes Mellitus

**DOI:** 10.7759/cureus.45650

**Published:** 2023-09-20

**Authors:** Aysima Bulca Acar

**Affiliations:** 1 Family Medicine, University of Health Sciences Antalya Training and Research Hospital, Antalya, TUR

**Keywords:** screening for diabetes, periodic health examination, family medicine, family history of diabetes, diabetes mellitus

## Abstract

Purpose: This study aims to evaluate the presence and risk of diabetes mellitus (DM) and the factors affecting the risk in those who visited the family medicine outpatient clinic.

Methods: The present study included adult patients who presented to the outpatient clinic for periodic health examination between February 4, 2022, and April 4, 2022, and who had no known history of DM and were eligible for screening. Anthropometric measurements of the participants were made and their clinical and familial histories were taken in relation to DM. HbA1c and fasting blood glucose (FBG) tests were conducted for each participant.

Results: A total of 125 participants, 87 (69.6%) women and 38 (30.4%) men, were included in the study, and five (4%) participants had diabetes. The analysis of the independent risk factors associated with diabetes by multivariate logistic regression analysis revealed that the presence of DM in the family increased the risk of having HbA1c ≥ 5.7% (OR: 3.441; 95% CI: 1.381-8,574; p=0.008). Among women, the waist circumference being > 95 cm was determined as a discriminating factor for HbA1c ≥ 5.7% (sensitivity: 61.54% and specificity: 68.85%).

Conclusion: Accurate patient-centered risk assessments by family physicians can lead to positive lifestyle modifications in patients. For this purpose, family physicians should evaluate the patients for diabetes and its associated risk factors and encourage them to take measures in order to prevent diabetes.

## Introduction

Diabetes mellitus (DM) is a chronic metabolic disorder characterized by the presence of glucose concentrations higher than a threshold blood glucose concentration, usually occurring over many years and caused by the failure of the β-cells of the pancreas to secrete enough insulin to meet the demands of target tissues [1]. It is classified into four groups: type 1, type 2, gestational DM, and other specific types. Firstly, insulin deficiency in type 1 DM occurs more acutely in children and adolescents due to the destruction of pancreatic β-cells. Insulin resistance and defects in insulin secretion are quite evident in type 2 DM, which accounts for approximately 90-95% of all cases of diabetes. Gestational DM, on the other hand, refers to the type of diabetes that occurs during pregnancy, while other specific types refer to hyperglycemia resulting from a number of disorders eventually impacting the pancreas [2].

The diabetes-related burden of disease is increasing day by day. As reported by the International Diabetes Federation, the number of adults with diabetes was 537 million (20-79 years old) worldwide as of 2021 and this figure is expected to have reached 643 million by 2030 [3]. Another study has reported that diabetes may have affected 693 million adults by 2045 [4]. The rate of conversion to type 2 DM in individuals in the prediabetes period is between 5% and 10% per year [5].

According to studies specifically evaluating the cases in Türkiye, diabetes is prevalent in 13.7% of the population in a way that impacts approximately 7.5 million people. The fact that we have recently reached the incidence figures of diabetes, actually predicted by the World Health Organization for 2030, reveals that more critical actions need to be taken to cope with diabetes. Given that nearly 25% of Türkiye’s health expenditures are incurred by diabetes and related diseases, it seems clear that coping with diabetes is of great importance in that DM brings a considerable burden to the national economy in addition to that on people’s general well-being [6,7].

Type 2 DM mostly occurs after the age of 30; however, as a result of the increased obesity, the prevalence of cases of type 2 DM that occur in childhood or adolescence, has risen especially in the last 10-15 years. It is known that genetic predisposition plays an important role in this regard. As the genetic density in the family increases, so does the risk of diabetes in the next generations, as a consequence of which the disease starts to appear at earlier ages. Such patients are often obese or overweight. The Turkish Society of Endocrinology and Metabolism (TEMD) emphasizes for this purpose that all adults should be evaluated for type 2 DM by considering their demographic and clinical characteristics. In the Guideline for the Diagnosis, Treatment and Follow-up of DM Complications, which was previously published in 2020, it was recommended that people over the age of 40, regardless of weight, should be screened for type 2 DM every three years, and that people with a body mass index over 25 kg/m^2^ along with certain risk factors such as having a family history of DM and being hypertensive-dyslipidemic should be screened earlier and more frequently, while the age recommendation to start getting screened was updated to 35 in the latest guideline published in July 2022 [8].

Medical practice is based on a system that puts the individual at the center. Within this system, the physician is the person who watches the signs and symptoms of patients to offer them a solution based on medical knowledge and professional experience. On the way to this solution, the physician definitely needs objective data, including anthropometric measurements or various laboratory parameters in addition to questioning the patients about their signs, symptoms, and presence of chronic comorbidities or family history.

Thus, the aim of this study was to evaluate the presence and risks of type 2 DM and the factors affecting the risk in patients eligible for Type 2 DM screening among those visiting the outpatient clinic for periodic health examinations. Another aim of our study was to raise awareness of DM among patients.

## Materials and methods

Study design and participants

This cross-sectional and prospective study comprised all patients over the age of 40 years (the age limit was 40 years according to the valid guideline at the time of the data collection of the study; this criterion was updated to 35 years in July 2022), who were visiting the Family Medicine Outpatient Clinic of the Health Sciences University, Antalya Training and Research Hospital for periodic health examination, without a known diagnosis of DM and those aged 18 years and older with a body mass index (BMI) ≥ 25 kg/m^2^ as well as asymptomatic patients with risk factors such as having a family history of DM, leading a sedentary life (patients were asked whether they engaged in any physical activity during the day, and those who stated that they did not engage in any physical activity were defined as leading a sedentary life), and having dyslipidemia, as indicated in the TEMD Guidelines for the Diagnosis, Treatment and Follow-up of Diabetes Mellitus and Its Complications [8].

Data collection tool and the collection procedure

After the participants were informed about the study, informed consent forms were obtained from them. They were also asked about further characteristics such as gender, age, whether or not they were smoking, whether or not they had any chronic diseases, and whether or not they had regular physical activity; the collected information was then recorded. The height and weight of the participants were measured by the researcher to calculate the BMI values. Waist circumference was measured at the narrowest diameter between the arcus costarum and the spina iliaca anterior-superior of the pelvis. BMI was calculated as the person’s weight in kilograms divided by the square of height in meters. Abdominal obesity was considered ≥ 100 cm in men and ≥ 90 cm in women.

In a data collection period of two months, this study included 125 volunteer participants with no known DM who met the specified criteria for type 2 DM screening from among 400 participants who visited the outpatient clinic for periodic health examinations between February 4, 2022, and April 4, 2022. There were no non-volunteers during the data collection process.

In the process, fasting blood glucose (FBG) and HbA1c tests were ordered for 125 people who were eligible for screening. Those with the HbA1c level ≥ 6.5% and FBG ≥ 126 mg/dL were considered diabetic as specified in the relevant guidelines, while those whose HbA1c was between 5.7-6.4% and FBG between 100-125 mg/dL were considered at risk for DM.

Ethical approval

Prior to the study, approval was obtained from the Clinical Research Ethics Committee of the University of Health Sciences Antalya Training and Research Hospital as of 03.02.2022 with decision number 3/18. The study was conducted in accordance with the Declaration of Helsinki.

Statistical analysis

Data were analyzed using the IBM Statistical Package for the Social Sciences (SPSS) 23.0 package program (IBM Corp., Armonk, NY). The conformity of continuous variables to normal distribution was examined by the Shapiro-Wilk test. Categorical variables were presented as frequency (n) and percentage (%), and continuous variables were presented as mean, standard deviation, median, and interquartile range (IQR: 25th-75th percentile) and analyzed by using Pearson’s chi-square and Fisher’s Exact Test. Independent t-test was used for continuous variables that met the parametric test assumptions, whereas the Mann-Whitney U test was used for those that did not meet the parametric test assumptions. Receiver operating characteristic (ROC) analysis was made to determine the diagnostic performance and cut-off point of age and waist circumference parameters to reveal the presence of HbA1C ≥ 5.7%. The results were presented as the area under the curve (AUC), cut-off points, sensitivity, and selectivity values with 95% confidence intervals. The optimal cut-off point of the parameters was estimated with the Youden index. Multivariate logistic regression analysis was made for the purpose of determining the independent risk factors for the case of HbA1C ≥ 5.7%. Variables with p < 0.2 in univariate analyses were included in the multivariate model and analysis results were presented with odds ratio (OR) and at 95% confidence intervals. The statistical significance level was accepted as 0.05 in the study.

## Results

The mean age of the 125 participants, 87 (69.6%) women and 38 (30.4%) men, was 46.12±13 years, and five (4%) participants turned out to have diabetes (Table 1).

**Table 1:** General characteristics of the participants BMI: body mass index, DM: diabetes mellitus, FBG: fasting blood glucose Categorical variables were presented as frequency (n) and percentage (%), and continuous variables were presented as mean, standard deviation, median, and interquartile range (IQR: 25th-75th percentile).

Variables	All participants (n=125)
Age, mean ± SS	46.12±13
Gender, n (%)	
Male	38 (30.4)
Female	87 (69.6)
Weight (kg), median (IQR)	76 (67-90)
BMI, median (IQR)	28.06 (25.61-30.86)
Waist circumference, median (IQR)	96 (89-105)
Abdominal obesity, n (%)	79 (63.2)
Smoker, n (%)	43 (34.4)
Chronic disease, n (%)	39 (31.2)
Disease type, n (%)	
Hypertension	18 (14.4)
Hypothyroid	11 (8.8)
Rheumatic disease	7 (5.6)
Malignity	3 (2.4)
Reason for ordering tests, n (%)	
BMI ≥ 25 kg/m^2^	30 (24)
> 40 years	95 (76)
Family history of DM, n (%)	69 (55.2)
HbA1c, median (IQR)	5.5 (5.3-5.8)
DM status relative to HbA1c level, n (%)	
Non-diabetic	81 (64.8)
At risk (5.7-6.4%)	41 (32.8)
Diabetic (≥6.5%)	3 (2.4)
FBG, median (IQR)	90 (84-98)
DM status relative to FBG level, n (%)	
Non-diabetic	97 (77.6)
At risk (100-125 mg/dL)	25 (20)
Diabetic (≥126 mg/dL)	3 (2.4)
Having DM, n (%)	5 (4)

One of the five patients we identified as diabetic in this study had an HbA1c level of 9.5%.

The evaluation of the study sample according to their FBG results revealed that the mean age of those in the diabetic or at-risk group in terms of DM was significantly higher than that of the non-diabetic group (p=0.006). In addition, waist circumference values were found to be significantly higher in the at-risk or diabetic group (p=0.007) (Table 2).

**Table 2:** The relationship between diabetes and patient characteristics according to fasting blood glucose Independent t-test, Mann-Whitney U test, Pearson chi-square test, Fisher’s exact test. BMI: body mass index, DM: diabetes mellitus Categorical variables were presented as frequency (n) and percentage (%), and continuous variables were presented as mean, standard deviation, median, and interquartile range (IQR: 25th-75th percentile). *p < 0.05 (significant)

Variables	Non-diabetic (n=97)	At-risk or diabetic (n=28)	P
Age, mean ± SS	44.43±13.19	51.96±10.61	0.006*
Gender, n (%)			
Male	25 (25.8)	13 (46.4)	0.036*
Female	72 (74.2)	15 (53.6)	
BMI, median (IQR)	27.94 (25.07-30.42)	29.27 (26.58-33.24)	0.100
Waist circumference, median (IQR)	94 (88-103)	100.5 (94.5-107.5)	0.007*
Abdominal obesity, n (%)	58 (59.8)	21 (75)	0.142
Smoker, n (%)	34 (35.1)	9 (32.1)	0.775
Chronic disease, n (%)	29 (29.9)	10 (35.7)	0.558
Hypertension	11 (11.3)	7 (25)	0.122
Hypothyroid	10 (10.3)	1 (3.6)	0.453
Family History of DM, n (%)	52 (53.6)	17 (60.7)	0.505

When evaluated considering the HbA1c level, the high-risk or diabetic group of participants appeared to have significantly higher mean age (p=0.002), higher tendency of developing abdominal obesity (p=0.044), and more family history of DM alike (p=0.011) (Table 3).

**Table 3:** Association of patient characteristics with diabetes in relation to HbA1c level Independent t-test, Mann-Whitney U test, Pearson chi-square test, Fisher’s Exact test. BMI: body mass index, DM: diabetes mellitus Categorical variables were presented as frequency (n) and percentage (%), and continuous variables were presented as mean, standard deviation, median, and interquartile range (IQR: 25th-75th percentile). *p < 0.05 (significant)

Variables	Non-diabetic (n=81)	At-risk or diabetic (n=44)	P
Age, mean ± SS	43.49±13.9	50.95±9.53	0.002*
Gender, n (%)			
Male	20 (24.7)	18 (40.9)	0.060
Female	61 (75.3)	26 (59.1)	
BMI, median (IQR)	28.2 (25.33-30.41)	28.01 (25.66-32.74)	0.326
Waist circumference, median (IQR)	94 (88-101)	102.5 (92.5-107.5)	0.002*
Abdominal obesity, n (%)	46 (56.8)	33 (75)	0.044*
Smoker, n (%)	29 (35.8)	14 (31.8)	0.654
Chronic disease, n (%)	21 (25.9)	18 (40.9)	0.084
Hypertension	5 (6.2)	13 (29.5)	<0.001*
Hypothyroid	8 (9.9)	3 (6.8)	0.745
Family history of DM, n (%)	38 (46.9)	31 (70.5)	0.011*

Independent risk factors associated with diabetes were analyzed by multivariate logistic regression analysis. It was determined that the presence of DM in the family increased the risk of having HbA1c ≥ 5.7% (OR: 3.441; 95% CI: 1.381-8,574; p=0.008) (Table 4).

**Table 4:** Determination of factors affecting HbA1c ≥ 5.7% by multivariate logistic regression analysis DM: diabetes mellitus, HT: hypertension Variables with p < 0.2 in univariate analyses were included in the multivariate model and analysis results were presented with odds ratio (OR) and at 95% confidence intervals (CI).

Variables	OR (95% CI)	P
Age	1.055 (1.016-1.096)	0.006
Male	1.181 (0.462-3.019)	0.728
Waist circumference	1.038 (0.998-1.078)	0.060
HT	2.49 (0.676-9.169)	0.170
Family history of DM	3.441 (1.381-8.574)	0.008

Table 5 presents the results of the ROC analysis for age and waist circumference in distinguishing the presence of HbA1c ≥ 5.7% in participants. Age and waist circumference were found to be discriminating factors for HbA1c ≥ 5.7% in women, whereas waist circumference was not found to be a discriminating factor in men. With the Youden index, the optimal cut-off point for age was found to be >42 (sensitivity: 81.82% and specificity: 48.15%), and for waist circumference in women was >95 (sensitivity: 61.54% and specificity: 68.85%) (Table 5).

**Table 5:** Distinctive role of age and waist circumference measurements in predicting the potential of HbA1c ≥ 5.7% The results were presented as area under the curve (AUC), cut-off points, sensitivity, and selectivity values with 95% confidence intervals (CI). The optimal cut-off point of the parameters was estimated with the Youden index. *p < 0.05 (significant)

Variables	AUC (95% CI)	P	Cut-off value	Sensitivity (%)	Specificity (%)
Age	0.680 (0.590-0.760)	<0.001*	>42	81.82	48.15
Waist circumference	0.665 (0.575-0.747)	0.002*	>100	54.55	74.07
Female	0.654 (0.544-0.752)	0.028*	>95	61.54	68.85
Male	0.579 (0.408-0.737)	0.415	-	-	-

**Figure 1:**
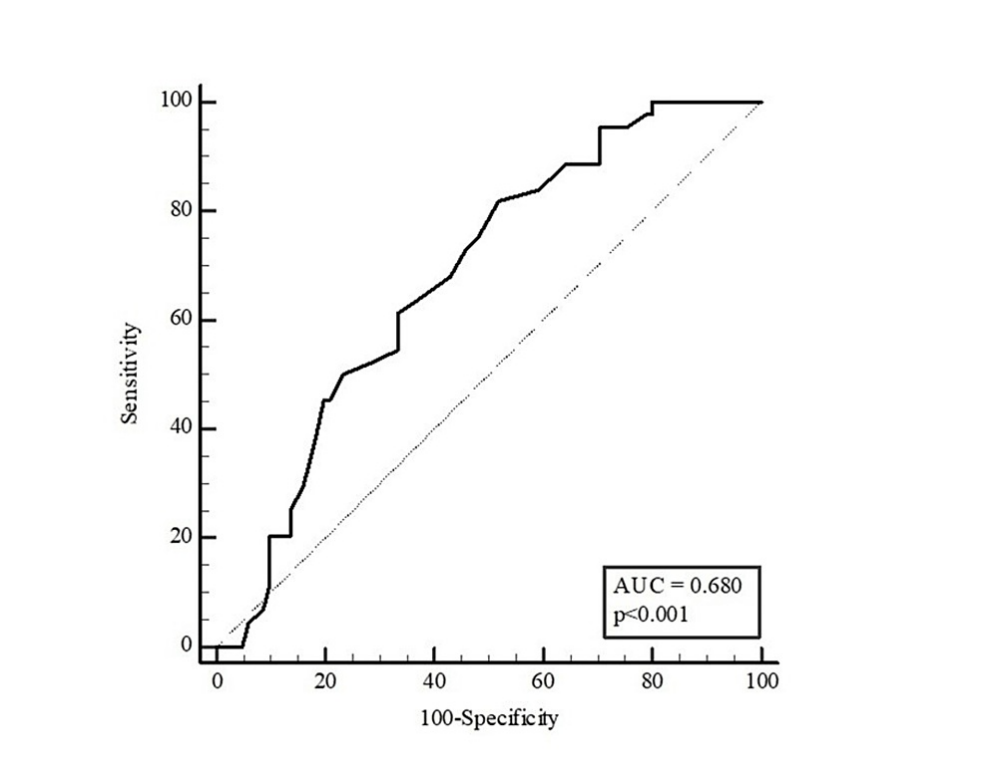
ROC curve for age to distinguish the potential of HbA1c ≥ 5.7% in participants AUC: area under the curve, (95% confidence intervals: 0.590-0.760; p<0.001), ROC: receiver operating characteristic

As a result of the ROC analysis, the AUC for age was estimated as 0.680 (95% CI: 0.590-0.760; p<0.001) (Figure 1).

**Figure 2:**
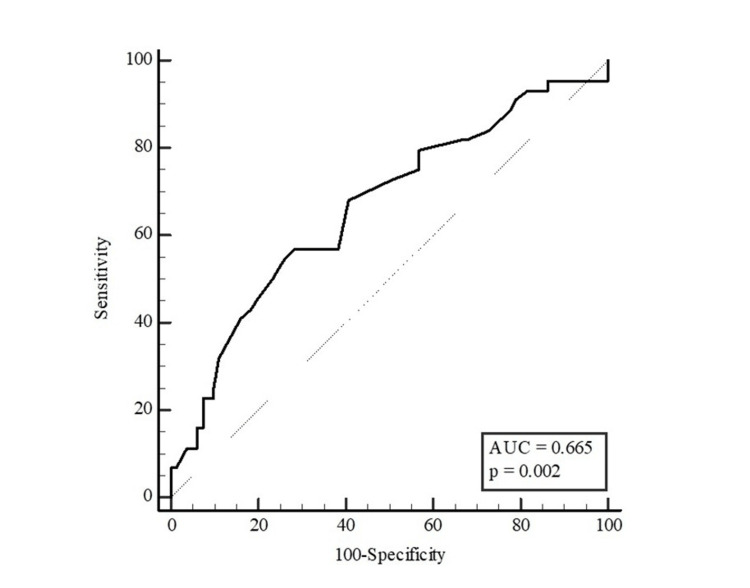
ROC curve for waist circumference to distinguish the potential of HbA1c ≥ 5.7% in participants AUC: area under the curve, (95% confidence intervals: 0.575-0.747; p=0.002), ROC: receiver operating characteristic

**Figure 3:**
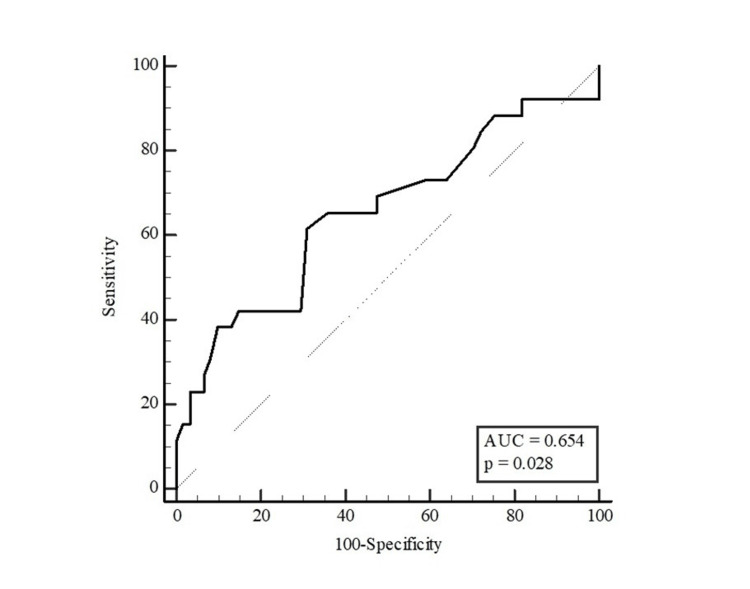
ROC curve for waist circumference to distinguish the potential of HbA1c ≥ 5.7% in women AUC: area under the curve, (95% confidence intervals: 0.544-0.752; p=0.028), ROC: receiver operating characteristic

**Figure 4:**
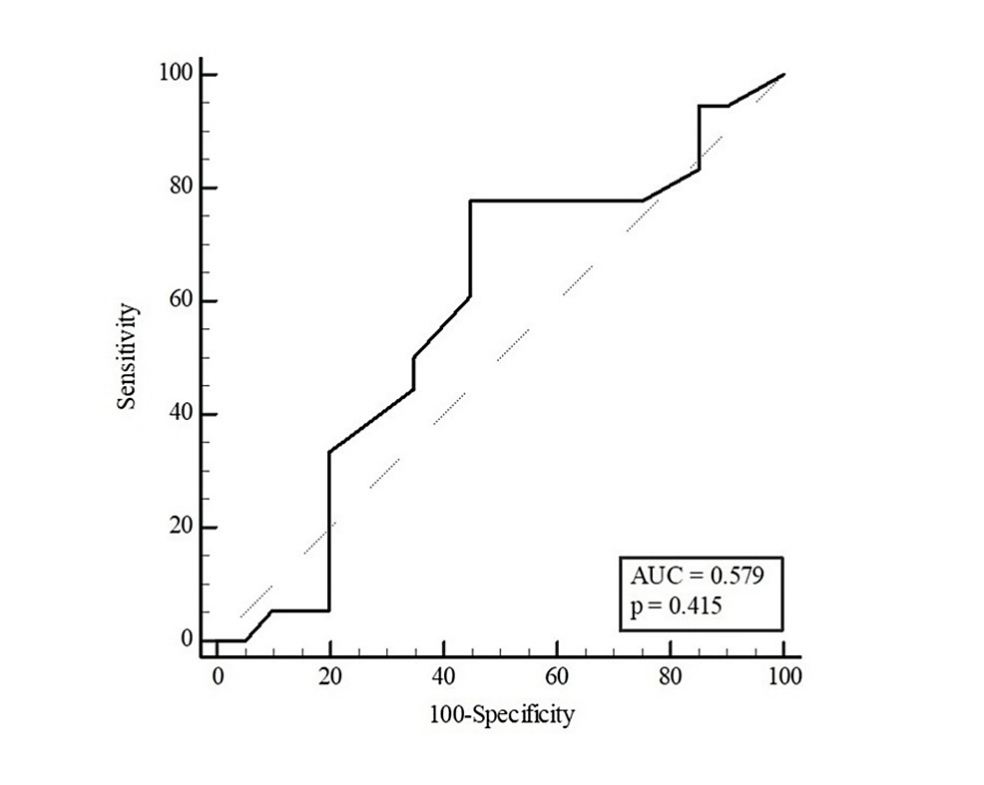
ROC curve for waist circumference to distinguish the potential of HbA1c ≥ 5.7% in men AUC: area under the curve, (95% confidence intervals: 0.408-0.737; p=0.415), ROC: receiver operating characteristic

The AUC for waist circumference was 0.665 for all participants (95% CI: 0.575-0.747; p=0.002; Figure 2), 0.654 for women (95% CI: 0.544-0.752; p=0.028; Figure 3) and 0.579 for men (95% CI: 0.408-0.737; p=0.415; Figure 4).

## Discussion

Conducted to evaluate the presence of DM, risk levels, and factors affecting the risk in people who visited the outpatient clinic for periodic health examinations, this study revealed that 4% of the participants actually had diabetes. The evaluation in relation to HbA1c levels also revealed that a family history of DM increased the risk of having DM. In addition, advanced age was found to be associated with an increased risk of diabetes.

Being an important public health concern worldwide, type 2 DM can remain asymptomatic for many years, despite being in a progressive course. Consequently, the risk of chronic complications of the disease and the related burden of disease increase alike [9].

Considering its acute complications, DM can not only cause diabetic ketoacidosis and hyperglycaemic hyperosmolar nonketotic hyperosmolar coma, but can also result in devastating macrovascular complications such as coronary artery disease, cerebrovascular disease, and peripheral artery disease, as well as a number of microvascular complications such as retinopathy, nephropathy, and neuropathy associated with increased mortality and decreased quality of life [4,10].

In this sense, screening approaches have been developed for early diagnosis of the disease. Nevertheless, type 2 DM screening is also reported not to decrease mortality in the 10-year period, but has positive effects on mortality after 23-30 years [11].

Currently, there is still no clarity on the impact of screening for type 2 DM on the mortality process. A 30-year study of 576 participants with impaired glucose tolerance in China has reported that those who agreed to an intervention to change their lifestyles, that is, changes including diet and exercise, presented a significantly delayed onset of diabetes, less cardiovascular disease, fewer microvascular complications, and fewer all-cause deaths [12]. No assessment was made in our study regarding mortality, but the age-related optimal cut-off point for DM risk was found to be 42. Our study was based on the 2020 TEMD Guidelines and the age limit for screening people without risk factors was indicated as 40 years in the guideline applicable in that period. However, in the new guideline published in July 2022, this limit was updated to 35 years of age [8]. The reason why we found the optimal cut-off point for age as 42 may be that 76% of our study sample consisted of people over the age of 40.

The evaluation of the HbA1c levels revealed that a family history of DM increased the risk of developing DM with an OR of 3.44, though no significant correlation was found between FBG levels and family history. In this connection, a study conducted by Geetha et al. with 215 diabetic patients reported that 90.9% of those aged between 21 and 30 years at the onset of diabetes had a family history of DM [13]. Despite the fact that a family history of DM is a well-recognized risk factor for type 2 DM by medical authorities, the acceptance of this risk by patients is essential for the development of appropriate health behaviors in patients. Moreover, a study of participants without known DM but with a family history of DM reported that family history was recognized as a risk factor by only 55% of participants [14].

A meta-analysis suggested that a family history of DM increases the risk of type 2 DM with an OR of 6.14 [15]. The same meta-analysis reported that the prevalence of type 2 DM is higher in those aged ≥ 40 years, those with BMI ≥ 25 kg/m^2^, and those with systolic blood pressure ≥ 140 mmHg [15].

It is also known that 70% of individuals with type 2 DM develop hypertension (HT), whose development is 2.5 times higher in individuals with known HT [16]. Since HT and DM frequently coexist, this co-occurrence is associated with increased mortality [16]. When evaluated according to HbA1c level, the presence of HT was found to be significantly associated with DM risk in support of this information. Research also indicates that HbA1c levels are associated with increased health-related risks and mortality [17,18]. The fact that the HbA1c level of one of the patients who was found to be diabetic in this study was 9.5% supports the need to increase the level of awareness of both clinicians and patients in terms of risks in the early period. Clinicians’ lifestyle recommendations and pharmacologic treatment approaches may prevent or delay the onset of diabetes in individuals at risk, such as those with a family history, HT, and advanced age. Lifestyle modification is reported to be equally effective in preventing the disease in people at high risk for type 2 DM, regardless of family history [19]. Therefore, positive lifestyle modification proves to prevent the development of type 2 DM in both cases, with or without a family history.

In our study, waist circumference values were found to be significantly higher in the evaluation by considering both the FBG and HbA1c levels. Detailed analyses showed that waist circumference was a discriminating factor in determining HbA1c ≥ 5.7% in women, whereas it was not found to be a discriminating factor in men. A study conducted in Jordan reported that a waist circumference > 92 cm and waist-to-height ratio > 0.60 in women and a waist circumference > 100 cm and waist-to-height ratio > 0.57 in men increased the likelihood of DM and HT by 3-5 times in women and 2-4 times in men [20].

In our study, the appropriate cut-off point for waist circumference in women with DM risk was 95 cm. The waist-to-height ratio is said to be crucial in predicting cardiometabolic abnormalities by taking into account both central fat accumulation and individual differences in height [21].

Limitations

One of the limitations of the current study is that no assessment of waist-to-height ratio was made in our study.

No correlation was found in our study between BMI and risk levels, a result which may be explained by the fact that our study sample was limited to 125 participants, and the BMI values of the participants who came for the periodic health screening were distributed in limited ranges.

The present study included a small sample size of 125 patients who presented to the Family Medicine Outpatient Clinic of the Health Sciences University, Antalya Training and Research Hospital for periodic health examination, so our results can be minimally generalized to the rest of the population.

## Conclusions

Continuous education of healthcare professionals and patients is essential to reduce the risk of acute complications of diabetes and to prevent long-term chronic complications affecting the retinal, renal, neural, cardiac, and vascular systems and related treatment costs. The biggest responsibility falls to family physicians offering primary health care and acting as a bridge between advanced health services.

The development of appropriate health behaviors can only be possible through appropriate lifestyle modifications. However, lifestyle modifications can only be tangible products of correct patient-centered personal risk assessments. For this purpose, family physicians should evaluate the patients for diabetes and its associated risk factors and encourage them to take measures in order to prevent diabetes.
